# The ESCRT-III complex is required for nuclear pore complex sequestration and regulates gamete replicative lifespan in budding yeast meiosis

**DOI:** 10.1080/19491034.2020.1812872

**Published:** 2020-09-06

**Authors:** Bailey A. Koch, Elizabeth Staley, Hui Jin, Hong-Guo Yu

**Affiliations:** Department of Biological Science, The Florida State University, Tallahassee, FL, USA

**Keywords:** ESCRT-III, lem-domain protein, nuclear pore complex, nuclear envelope, meiosis, replicative lifespan, cellular aging

## Abstract

Cellular aging occurs as a cell loses its ability to maintain homeostasis. Aging cells eliminate damaged cellular compartments and other senescence factors via self-renewal. The mechanism that regulates cellular rejuvenation remains to be further elucidated. Using budding yeast gametogenesis as a model, we show here that the endosomal sorting complex required for transport (ESCRT) III regulates nuclear envelope organization. During gametogenesis, the nuclear pore complex (NPC) and other senescence factors are sequestered away from the prospore nuclei. We show that the LEM-domain protein Heh1 (Src1) facilitates the nuclear recruitment of ESCRT-III, which is required for meiotic NPC sequestration and nuclear envelope remodeling. Furthermore, ESCRT-III-mediated nuclear reorganization appears to be critical for gamete rejuvenation, as hindering this process curtails either directly or indirectly the replicative lifespan in gametes. Our findings demonstrate the importance of ESCRT-III in nuclear envelope remodeling and its potential role in eliminating senescence factors during gametogenesis.

## Introduction

Cellular aging occurs as a cell’s ability to maintain homeostasis deteriorates, affecting both the chronological age, the cell can attain and its replicative lifespan. Replicative lifespan refers to the number of daughter cells a mother cell generates before death; dysregulation of replicative lifespan is characteristic of diseases of aging and cancer [1,2]. The resetting of replicative potential is best characterized in budding yeast, *S. cerevisiae* [3–5]. Vegetative yeast cells undergo asymmetric cell division, in which the mother cell retains aging factors such as old or damaged cellular compartments, protein aggregates, and extra chromosomal rDNA circles, allowing the daughter cell to inherit a full replicative lifespan [6–10]. Interestingly, self-renewal has also been observed during gametogenesis in budding yeast [11], in which nuclear divisions are symmetrical. However, the molecular mechanism regulating gametogenic self-renewal remains to be determined.

A key factor in mitigating cellular aging is proper nuclear trafficking between the cytoplasm and nucleoplasm [12]. The eukaryotic nucleus is enclosed by two membranes that separate these regions, forming a selective barrier. The outer nuclear membrane (ONM) is continuous with the endoplasmic reticulum (ER), whereas the inner nuclear membrane (INM) interacts with the nucleoplasm [13]. Embedded within the nuclear envelope are nuclear pore complexes (NPCs). NPCs form a ~ 50 MD protein channel that is composed of concentric inner, outer, and membrane ring assemblies that scaffold a central transport channel, cytosolic filaments, and nuclear basket [14,15]. These NPC domains are constructed from modular subcomplexes of proteins termed nucleoporins or nups [15,16]. NPCs are the primary method of nuclear trafficking, imposing a diffusion barrier to molecules larger than ~40 kD, while also providing binding sites for the rapid and selective transport of molecules containing nuclear import and export signals [17–19]. The semipermeable barrier provided by NPCs aids in maintaining nuclear integrity and nuclear envelope homeostasis. Loss of nuclear compartmentalization through NPC damage or assembly defects is associated with cellular aging [12,20,20,21].

Approximately 100 NPCs are formed during budding yeast mitosis [22]. NPC biogenesis likely occurs through an inside-out evagination of the INM followed by membrane fusion with the ONM [23], resulting in nuclear envelope holes containing NPC assembly intermediates that develop into mature NPCs [24,25]. Due to the crucial function of NPCs in maintaining cellular health, a surveillance mechanism is in place to ensure proper biogenesis [21]. Surveillance of NPC biogenesis is mediated by the LEM (Lap2-emerin-MAN1) family of integral INM proteins, Heh1 (also called Src1; for consistency, we use Heh1 hereafter) and Heh2, in addition to the endosomal sorting complex required for transport (ESCRT) III subunits Chm7, Snf7, and the AAA-ATPase Vps4 which function to destabilize and clear defective NPC assembly intermediates [21,25]. It has been proposed that Heh1 is required for the focal accumulation of Chm7 at the nuclear envelope in genetic backgrounds where NPC assembly is inhibited and ESCRT-III activation at the nuclear envelope is likely through a direct interaction between Snf7 and Chm7 in conjunction with Heh1 and Heh2 [24,25].

In the absence of the ESCRT-III surveillance mechanism, malformed NPCs in mitosis accumulate in a storage of improperly assembled nuclear pore complexes compartment, or SINC [21]. The SINC, containing misassembled or damaged NPCs, is not passed on to daughter cells but instead remains in the mother with other unwanted materials, resulting in a daughter cell free of senescence factors [21,26,27]. While there is sufficient evidence for ESCRT-III and its associated proteins function in NPC quality control during budding yeast mitosis, little is known about the regulation of NPCs and cellular aging in meiosis. In contrast to mitosis, meiosis consists of two rounds of chromosome segregation resulting in four gametes, also referred to as spores in budding yeast [28]. Unlike the asymmetric division in vegetative yeast cells, meiotic cells reset aging symmetrically such that all gametes have a renewed replicative lifespan regardless of the age of the mother cell [11,29]. Importantly, senescence factors originally present in the mother cell, such as protein aggregates and extrachromosomal rDNA circles, are no longer present in the newly formed gametes [11,29–31].

We show here that nucleoporins are selectively sequestered away from the newly formed gametes during meiosis II, and this process is regulated by the ESCRT-III complex. In the absence of ESCRT-III function, gametes exhibit a reduced replicative lifespan, suggesting that ESCRT-III-mediated nuclear envelope remodeling is crucial for gamete rejuvenation. Our work provides evidence for a potential ESCRT-III mediated meiotic mechanism for sequestering unwanted nuclear materials in a mother cell, resulting in the renewal of the newly formed gametes.

## Materials and Methods

### Yeast strains and plasmids used in this study

Yeast strains used in this study are isogenic to the SK1 background and are listed in Table 1. To tag the C-terminus of Hta1, Heh1, Vps4, Nup49, Pom34, and other nucleoporins with either GFP or mApple, we used a standard PCR-based homologous recombination method [32]. Similarly, we used this technique to tag Vps4 with V5 and an auxin-inducible degron (AID) in tandem to generate Vps4-V5-AID and to generate Vps4-GFP. These tagged alleles are the only functional copies of the corresponding genes in the yeast genome. A comparable PCR-based method was used to replace the *HEH1, HEH2, CHM7*, and *SPO21* open reading frames with either a KanMX4 or Hygromycin-B cassette to generate gene deletions. Correct transformations were further confirmed by colony-based diagnostic PCR. Primers are listed in Table 2.

**Table 1.** Yeast strains used in this study.

Strain	Background	Genotype	Experiment
HY6229	SK1	*MATa/MATα, his3∆200, leu2-k, ura3, lys2, ho::LYS2, POM34-GFP//his3∆200, leu2-k, ura3, lys2, ho::LYS2, POM34-GFP, HTA1-mApple*	Figures 1b, 4e, 4f, 4g, 4i, and 6
HY6279	SK1	*MATa/MATα, his3∆200, leu2-k, ura3, lys2, ho::LYS2, POM34-GFP, NUP49-mApple//his3∆200, leu2-k, ura3, lys2, ho::LYS2, POM34-GFP, NUP49-mApple*	Figure 1c
HY6200	SK1	*MATa/MATα, his3∆200, leu2-k, ura3, lys2, ho::LYS2, HTA1-mApple//his3∆200, leu2-k, ura3, lys2, ho::LYS2, P_DMC1_-MPS3-NC*	Figure 2a
HY6695	SK1	*MATa/MATα, ho::LYS2, lys2, ura3, leu::hisG, his3::hisG, trp1::hisG, P_GAL_-NDT80::TRP1, ura3::P_GPD1_-GAL4.ER::URA3, P_GAL1_-GFP-Mps2//ho::LYS2, lys2, ura3, leu::hisG, his3::hisG, trp1::hisG, P_GAL_-NDT80::TRP1, ura3::P_GPD1_-GAL4.ER::URA3, P_GAL1_-GFP-Mps2, HTA1-mApple*	Figures 2b and 2c
HY6586-2	SK1	*MATa/MATα, his3∆200, leu2-k, ura3, lys2, ho::LYS2, heh1∆::KAN, POM34-GFP//his3∆200, leu2-k, ura3, lys2, ho::LYS2, heh1∆::KAN, POM34-GFP, HTA1-mApple*	Figures 3b, 3d, 3e, 4e, and 6
HY6356	SK1	*MATa/MATα, his3∆200, leu2-k, ura3, lys2, ho::LYS2, heh2∆::KAN, POM34-GFP//his3∆200, leu2-k, ura3, lys2, ho::LYS2, heh2∆::KAN, POM34-GFP, HTA1-mApple*	Figures 3c, 3d, and 3e
HY6389	SK1	*MATa/MATα, his3∆200, leu2-k, ura3, lys2, ho::LYS2, heh1∆::KAN, heh2∆::KAN, POM34-GFP, HTA1-mApple//his3∆200, leu2-k, ura3, lys2, ho::LYS2, heh1∆::KAN, heh2∆::KAN, POM34-GFP, HTA1-mApple*	Figures 3d and 3e
HY6541	SK1	*MATa/MATα, his3∆200, leu2-k, ura3, lys2, ho::LYS2, chm7∆::HB, POM34-GFP//his3∆200, leu2-k, ura3, lys2, ho::LYS2, chm7∆::HB, POM34-GFP, HTA1-mApple*	Figures 4a, 4e, 4f, 4i, and 6
HY6585	SK1	*MATa/MATα, his3∆200, leu2-k, ura3, lys2, ho::LYS2, heh1∆::KAN, chm7∆::HB, POM34-GFP, HTA1-mApple//his3∆200, leu2-k, ura3, lys2, ho::LYS2, heh1∆::KAN, chm7∆::HB, POM34-GFP, HTA1-mApple*	Figures 4b, 4e, 4g, 4i, and 6
HY6608	SK1	*MATa/MATα, his3∆200, leu2-k, ura3, lys2, ho::LYS2, spo21∆::KAN, POM34-GFP//his3∆200, leu2-k, ura3, lys2, ho::LYS2, spo21∆::KAN, POM34-GFP, HTA1-mApple*	Figures 4 C, 4E, 4 H, and 4I
HY6614	SK1	*MATa/MATα, his3∆200, leu2-k, ura3, lys2, ho::LYS2, spo21∆::KAN, chm7∆::HB, POM34-GFP//his3∆200, leu2-k, ura3, lys2, ho::LYS2, spo21∆::KAN, chm7∆::HB, POM34-GFP, HTA1-mApple*	Figures 4D, 4E, 4 H, and 4I
HY6724	SK1	*MATa/MATα, his3Δ200, leu2-k, ura3, lys2, ho::LYS2, P_DMC1_-HEH1-mApple, VPS4-GFP::HIS5//his3Δ200, leu2-k, ura3, lys2, ho::LYS2, P_DMC1_-HEH1-mApple, VPS4-GFP::HIS5*	Figure 5A
HY6663	SK1	*MATa/MATα, ho::LYS2, lys2, ura3, leu::hisG, his3::hisG, trp1::hisG, P_GAL_-NDT80::TRP1, ura3::P_GPD1_-GAL4.ER::URA3, P_DMC1_-TIR1::LEU2, VPS4-V5-AID::HIS5, POM34-GFP, HTA1-mApple//ho::LYS2, lys2, ura3, leu::hisG, his3::hisG, trp1::hisG, P_GAL_-NDT80::TRP1, ura3::P_GPD1_-GAL4.ER::URA3, P_DMC1_-TIR1::LEU2, VPS4-V5-AID::HIS5, POM34-GFP, HTA1-mApple*	Figures 5B, 5 C, 5D, 5 F, 5 G, 5 H, 5I and 6
HY6644	SK1	*MATa/MATα, ho::LYS2, lys2, ura3, leu::hisG, his3::hisG, trp1::hisG, P_GAL_-NDT80::TRP1, ura3::P_GPD1_-GAL4.ER::URA3, POM34-GFP, HTA1-mApple//ho::LYS2, lys2, ura3, leu::hisG, his3::hisG, trp1::hisG, P_GAL_-NDT80::TRP1, ura3::P_GPD1_-GAL4.ER::URA3, POM34-GFP, HTA1-mApple*	Figures 5B, 5E and 5I
HY6705	SK1	*MATa/MATα, ho::LYS2, lys2, ura3, leu::hisG, his3::hisG, trp1::hisG, P_GAL_-NDT80::TRP1, ura3::P_GPD1_-GAL4.ER::URA3, P_DMC1_-TIR1::LEU, VPS4-V5-AID, heh1∆::KAN, POM34-GFP, HTA1-mApple//ho::LYS2, lys2, ura3, leu::hisG, his3::hisG, trp1::hisG, P_GAL_-NDT80::TRP1, ura3::P_GPD1_-GAL4.ER::URA3, P_DMC1_-TIR1::LEU2, VPS4-V5-AID, heh1∆::KAN, POM34-GFP, HTA1-mApple*	Figures 5B, 5 F, 5 G, 5 H and 5I

**Table 2.** Primers used in this study.

Primer Name	Sequence
POM34-tagF	GCAAATATGCATATATGATGAACTCACAGTCCCCAAGGGGGAAAATAGCGGCCGCTCTAGAACTAGT
POM34-tagR	TATATAGCTATGGAAAGTATTAAATGTTTTTTTGCTGTTTTCCCCCTCGAGGTCGACGGTA
HTA1-tagF	GTTGCCAAAGAAGTCTGCCAAGGCTACCAAGGCTTCTCAAGAATTAGCGGCCGCTCTAGAACTAGTGG
HTA1-tagR	GCAGTTTAGTTCCTTCCGCCTTCTTTAAAATACCAGAACCGATCTCCCCCTCGAGGTCGACGGTATCG
NUP49-tagF	GTTACATCAAAAAACGAAAACACTGGCATCATTGAGCATAGCGGCCGCTCTAGAACTAGT
NUP49-tagR	ACTTGTTATACGCACTATATAAACTTTCAGGGCGATTTACCCCCCTCGAGGTCGACGGTA
HEH1-deletionF	TCACCCTGAACGGAAATCAA
HEH1-deletionR	TTTCTTTCCTCCATGTGTCG
HEH2-deletionF	TGACAAGCACTATCTTCCAAAGT
HEH2-deletionR	GTATGCGTAGGGGAAGGGAT
HEH1-tagF	TCGATGAAAAGGTTAAACCGCAGATCCCGCAGTTACGGAAAtCAGGGGCATGATGTGACT
HEH1-tagR	TTCCGCCAATGTTGTTGTCAGTGGGTGATAACTAGAGAAAGCTCGTTTTCGACACTGGAT
CHM7-deletionF	AGTGCAGCGTTAGTAGAGACAATAAGAGGAGTTTTAAATTCTTAAACAGGGGCATGATGTGACT
CHM7-deletionR	TGCACAGGTCCTTCATTTGTATTTATCTTCAGATTATTCAATCTCTTTAATAGCTCGTTTTCGACACTGGAT
SPO21-deletionF	TCTGGGTTCAAGAATTCCTCAGA
SPO21-deletionR	GTTTCTTCGGCAACCCTGTA
VPS4-tagF	CTTGCTGAAGCAAGAACAGTTCACTAGAGAGATTTTGGTCAAGAAGGTAACGCGGCCGCTCTAGAACTAGTGG
VPS4-tagR	TATTTTCATGTACACAAGAAATCTACATTAGCACGTTAATCAATTGACCCCTCGAGGTCGACGGTATCG

**Figure 1.**
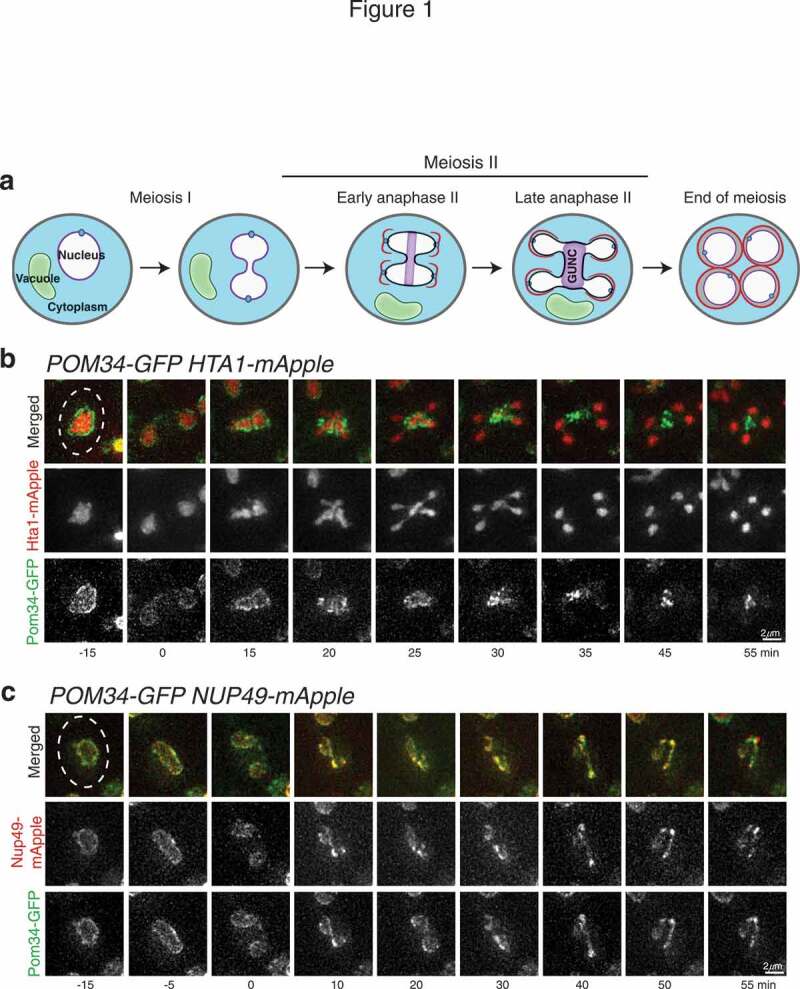
Sequestration of nucleoporins Pom34 and Nup49 to the GUNC during meiosis II. (a) Schematic representation of meiosis II in budding yeast. Note that nucleoporins (purple) are sequestrated to the GUNC and are largely excluded from the newly formed gamete nuclei. Blue dots represent the yeast spindle pole body; red lines, prospore membrane. (b) Time-lapse live-cell microscopy showing Pom34-GFP localization in meiosis II. Time 0 refers to the perceived start point of meiosis II based on nuclear morphology elucidated by the Hta1-mApple marker. Note that during anaphase II the majority of Pom34-GFP is aggregated to the midzone of the dividing nucleus. Projected images from 12 optical sections are shown. (c) Colocalization of Pom34-GFP and Nup49-mApple in meiosis II. Time-lapse live-cell microscopy was performed as in B. Note that Pom34 and Nup49 colocalize and are both largely excluded from the developing nuclei. Dashed lines show cell boundary.

**Figure 2.**
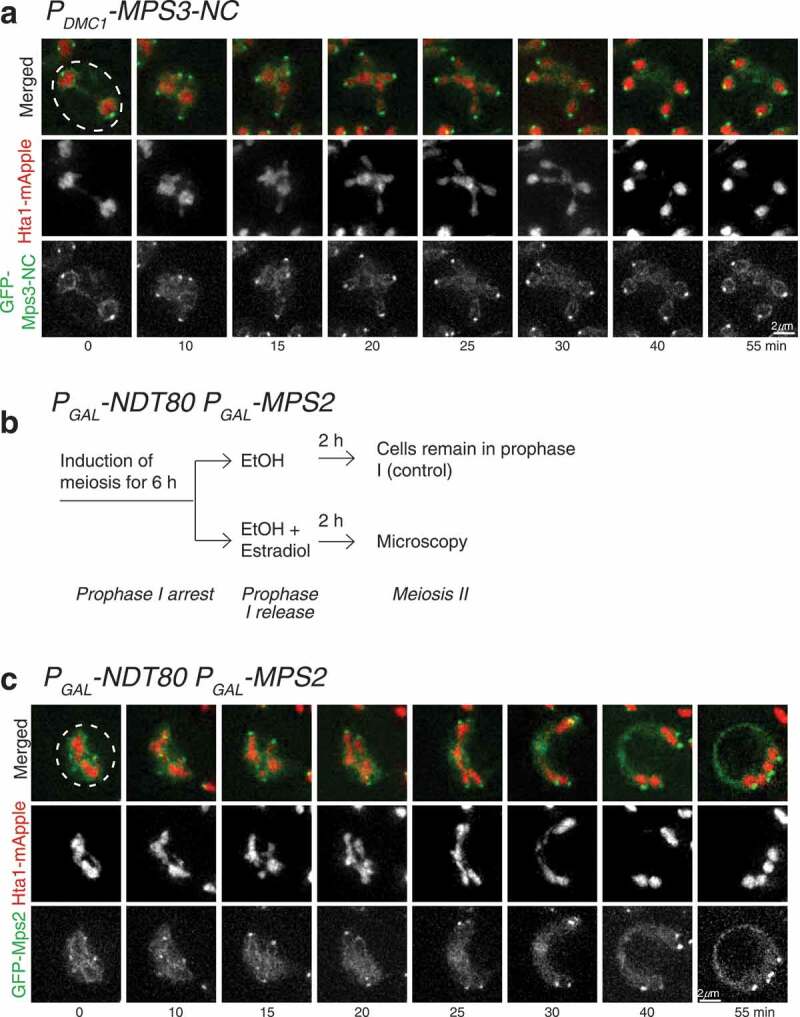
Localization of Mps3-NC and Mps2 in meiosis II. (a) Localization of Mps3-NC in meiosis. Time-lapse live-cell microscopy showing the localization of INM protein Mps3 during meiosis II. Time 0 refers to the perceived start point of meiosis II based on nuclear morphology as in Figure 1b. Note that during anaphase II GFP-Mps3-NC remains on the nuclear periphery and also forms distinct puncta, representing the spindle pole bodies. (b and c) Localization of Mps2 in meiosis. Schematic diagram shown in B illustrates the method of Mps2 overproduction in meiosis. Yeast cells were arrested at prophase I due to the lack of Ndt80, a meiosis-specific transcription factor required for expression of mid and later meiotic genes. Addition of estradiol led to production of Ndt80 and Mps2. Time-lapse live-cell microscopy was performed approximately 2 h after estradiol addition. Note that during anaphase II GFP-Mps2 localizes to both the spindle pole bodies and the nuclear periphery. Projected images from 12 optical sections are shown.

**Figure 3.**
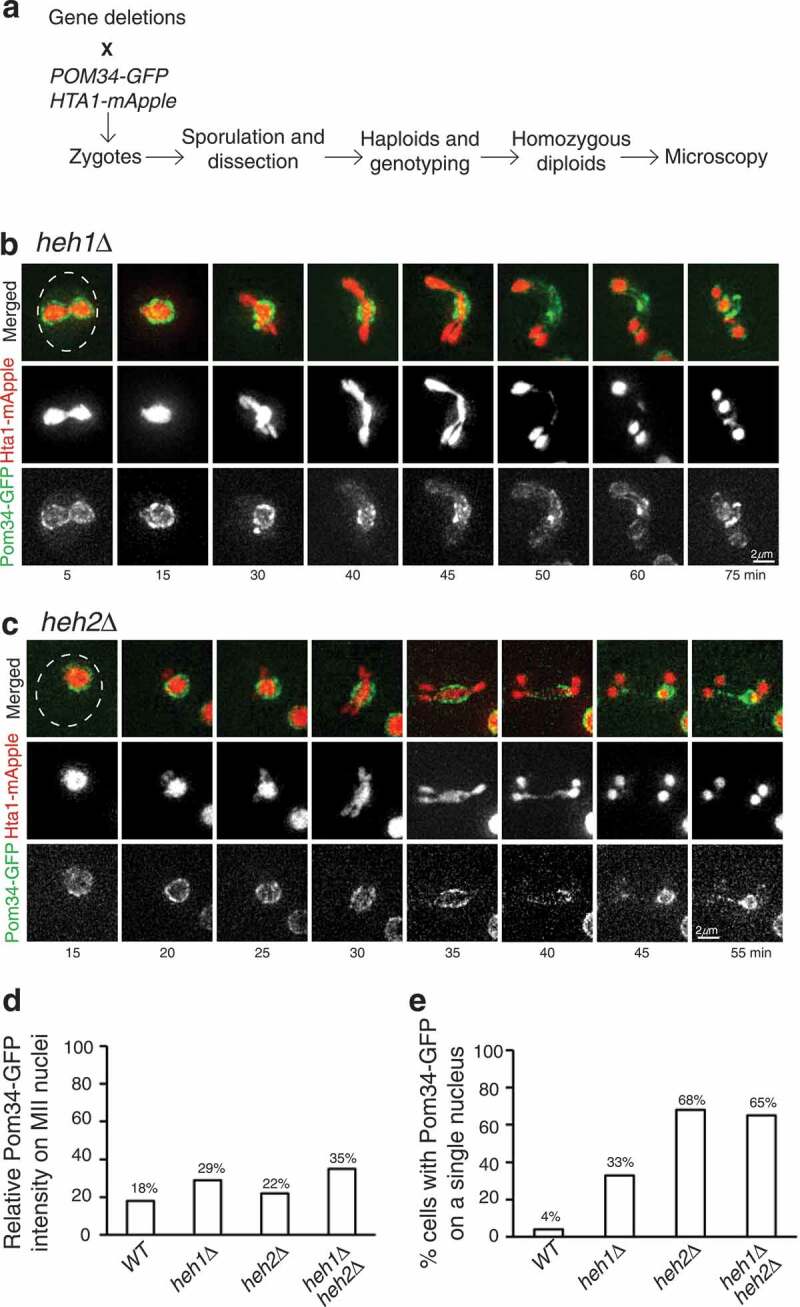
Heh1 and Heh2 regulate Pom34 sequestration in meiosis II. (a) Schematic diagram showing targeted genetic screen to identify genes that regulate Pom34 localization. Briefly, targeted gene deletions were crossed to a strain containing *POM34-GFP* and *HTA1-mApple*. Selective media allowed for the identification of heterozygous diploids, which were induced to undergo meiosis. Subsequent tetrads were dissected and genotyped, and zygotes for homozygous gene deletions were created and induced to undergo meiosis, followed by fluorescence microscopy. (b) Heh1 regulates Pom34-GFP sequestration. Time-lapse live-cell microscopy was performed as described in Figure 1b. Note that in the absence of Heh1, Pom34-GFP was no longer restricted to the GUNC. (c) Heh2 mediates Pom34-GFP distribution during meiosis II. Time-lapse microscopy was performed as described in Figure 1b. Note that in the absence of Heh2, Pom34-GFP encapsulated one of the Hta1 masses, in addition to localizing to the midzone. (d) Quantification of Pom34-GFP distribution to the meiotic nuclei 30 min into meiosis II in WT, *heh1Δ, heh2Δ*, and *heh1Δheh2Δ* cells. (e) Quantification of the percent of cells showing Pom34-GFP encapsulating a single Hta1-mApple mass in WT, *heh1Δ, heh2Δ*, and *heh1Δheh2Δ* yeast cells as shown in C. A minimum of 25 cells were analyzed for each strain over 3 biological replicates.

**Figure 4.**
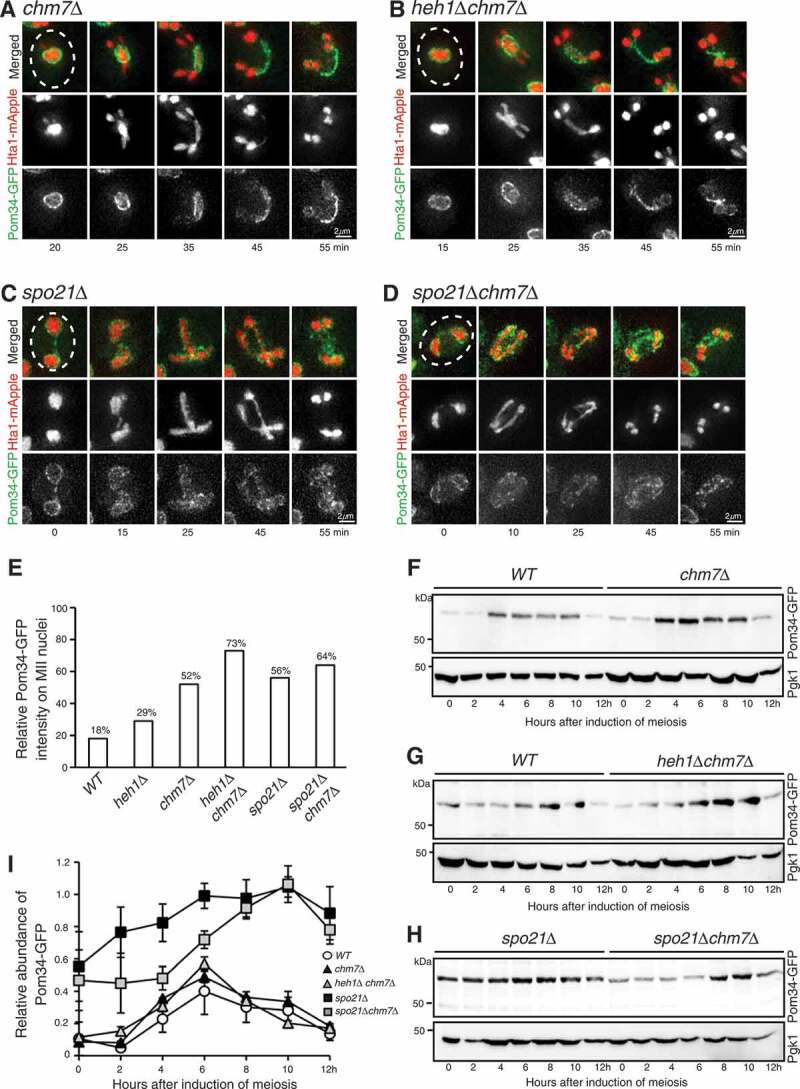
Chm7 regulates GUNC in budding yeast meiosis. (a) Representative images showing Pom34-GFP localization in a *chm7Δ* cell in meiosis. Time-lapse live-cell microscopy was performed as described in Figure 1b. Projected images from 12 optical sections are shown. (b) Representative images showing Pom34-GFP localization in a *heh1Δchm7Δ* double mutant cell. (c) Representative images showing Pom34-GFP localization in a *spo21Δ* cell. (d) Representative images showing Pom34-GFP localization in a *spo21Δchm7Δ* double mutant cell. (e) Quantification of Pom34-GFP distribution to the meiotic nuclei 30 min into meiosis II in WT, *heh1Δ, chm7Δ, heh1Δchm7Δ, spo21Δ*, and *spo21Δchm7Δ* cells. A minimum of 25 cells were analyzed for each strain over 3 biological replicates. One-way ANOVA analysis between *spo21Δ* and *spo21Δ chm7Δ* shows F ratio = 5.35601, p > 0.01; and between *heh1Δ, chm7Δ, heh1Δchm7Δ* F ratio = 8.2218, p < 0.01. (f-h) Pom34-GFP protein level in WT, *chm7Δ, heh1Δchm7Δ, spo21Δ*, and *spo21Δchm7Δ* cells. Cell aliquots were withdrawn at the indicated times after meiosis induction. Protein extracts were prepared for western blotting. An anti-GFP antibody was used to probe Pom34-GFP. The level of Pgk1 serves as a loading control. (i) Quantification of Pom34-GFP protein level in meiosis. Error bars represent the standard deviation from the mean of biological replicates (n = 3). Note that there is no discernable difference in Pom34-GFP protein levels between WT, *chm7Δ*, and *heh1Δchm7Δ* yeast cells, but deletion of *SPO21* alters Pom34-GFP protein level.

**Figure 5.**
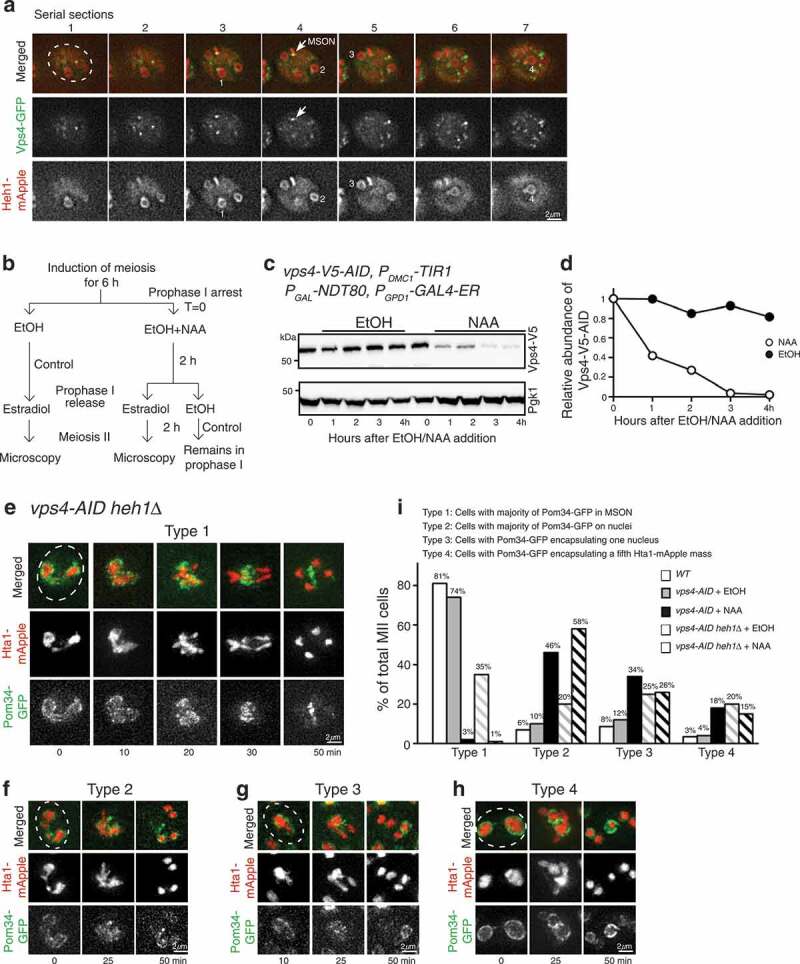
Vps4 localization and its role in Pom34 sequestration. (a) Localization of Vps4-GFP (green) in meiosis II. Time-lapse fluorescence microscopy was performed as in Figure 1b. Images from 7 continuous optical sections (z = 0.5 μm) were shown. Arrows indicate the localization of Vps4-GFP to the GUNC compartment. Note that Heh1-mApple (red) demarcates GUNC and the four nuclei, which are numerically labeled. (b) Schematic diagram showing the experimental procedure to deplete Vps4 in meiosis II. Addition of 1-napthylacetic acid (NAA) initiates Vps4 degradation through AID. Addition of estradiol releases cells from prophase I arrest. Time-lapse live-cell microscopy was performed approximately 2 h after estradiol addition to ensure cells were in meiosis II. Addition of ethanol serves as a negative control. (c and d) Depletion of Vps4-V5-AID in meiosis. Cells were prepared as described in B, and cell aliquots were withdrawn at indicated times. Time zero refers to the point of NAA addition. Protein extracts were prepared for western blotting. An anti-V5 antibody was used to probe Vps4-V5-AID. The level of Pgk1 serves as a loading control. Quantification of Vps4 protein abundance is shown in panel D. (e-h) Vps4 regulates Pom34-GFP localization and chromosome segregation. Cells were prepared as described in B. Projected images from 12 optical sections are shown. Four categories based on nuclear morphology were classified: Type 1, Pom34-GFP sequestered to GUNC as shown in E; Type 2, Pom34-GFP is concentrated on newly forming nuclei as shown in F; Type 3, Pom34-GFP encapsulates one of the four Hta1-mApple masses (nuclei) as shown in G; Type 4, Pom34-GFP encapsulates the fifth Hta1-mApple mass as shown in H. (i) Quantification of Pom34-GFP localization in cells with *WT, vps4-AID* with EtOH, *vps4-AID* with NAA, *vps4-AID heh1Δ* with EtOH, and *vps4-AID heh1Δ* with NAA. A minimum of 50 cells were analyzed for each strain background.

**Figure 6.**
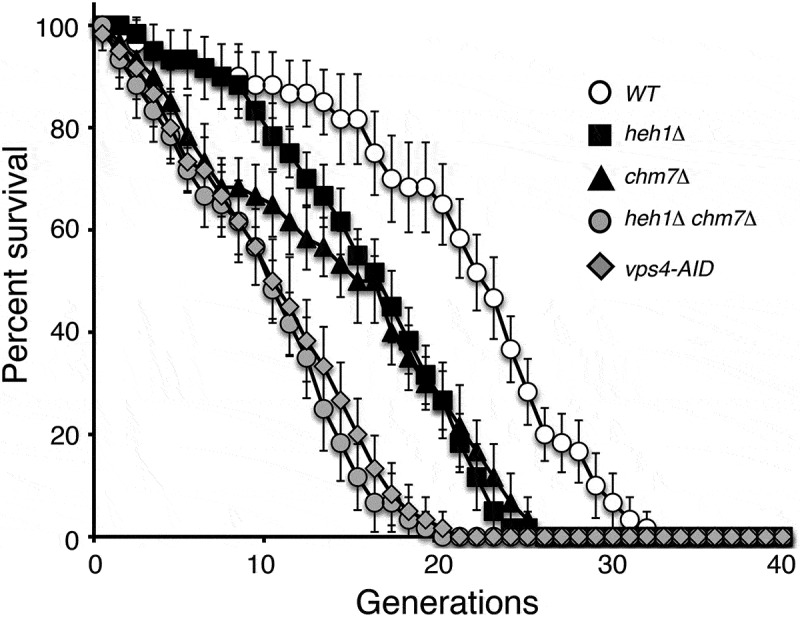
ESCRT-III and its associated factors regulate replicative lifespan in gametes. Replicative lifespan in wild-type and mutant gametes was determined by single-cell analysis using micromanipulation. Each cell analyzed was a gamete of the homozygous diploid originally analyzed, isolated at random from the population. The average life-span for *WT* gametes was 21.3, 17.3 for *heh1Δ*, 15.3 for *chm7Δ*, 9.9 for *heh1Δchm7Δ*, and 10.9 for *vps4-AID*. A total of 60 spores were assayed over 3 biological replicates for each strain. Error bars represent deviation from the mean.

Plasmids used in this study are included in Table 3. To sufficiently deplete Vps4-V5-AID, the plant F-box protein Tir1 is present in addition to auxin. We generated a *P_DMC1_-TIR1* (pHG273) construct to effectively overexpress *TIR1* in meiosis. Integration was achieved by linearizing pHG273 with StuI and integrating at the *URA3* locus by yeast transformation. Uracil – positive colonies were confirmed by colony-based diagnostic PCR. To overproduce Mps2 in meiosis II, we used the *GAL* promoter to construct *P_GAL_-GFP-MPS2* (pHG527) by placing the *MPS2* open reading frame under the control of the *GAL* promoter. We linearized plasmid pHG527 with StuI and integrated it at the endogenous *MPS2* locus by yeast transformation. Note that the endogenous *MPS2* remains intact and functional. Leucine-positive colonies were confirmed by diagnostic PCR. Similarly, we generated a *P_DMC1_-HEH1-mApple* (pHG742) construct to produce Heh1-mApple specifically in meiosis. Integration was achieved by linearizing pHG742 with XbaI and integrating at the *HEH1* locus. Note that the endogenous *HEH1* remains intact and functional. Positive colonies were confirmed by diagnostic PCR.

**Table 3.** Plasmids used in this study.

Plasmid Name	Description
pHG363	*P_DMC1_-GFP-MPS3-NC, LEU2*
pHG527	*P_GAL1_-GFP-MPS2, LEU2*
pHG496	*P_GAL1_-HEH1, LEU2*
pHG273	*P_DMC1_-TIR1, URA3*
pHG740	*P_DMC1_-TIR1, LEU2*
pHG742	*P_DMC1_-HEH1-mApple, LEU2*

**Table 4.** Gene deletions used in genetic screen.

Systematic name	Ploidy	Standard name
YML107C	MAT a	PML39
YMR129W	MAT a	POM152
YHR004 C	MAT a	NEM1
YER027C	MAT a	GAL83
YDR205W	MAT a	MSC2
YBR150C	MAT a	TBS1
YIL030C	MAT a	SSM4
YAL009W	MAT a	SPO7
YAR002W	MAT a	NUP60
YAR027W	MAT a	UIP3
YAR042W	MAT a	SWH1
YAR044W	MAT a	SWH1
YBL079W	MAT a	NUP170
YBR097W	MAT a	VPS15
YBR170C	MAT a	NPL4
YDL019C	MAT a	OSH2
YDL088C	MAT a	ASM4
YDL089W	MAT a	NUR1
YDL116W	MAT a	NUP84
YDR073W	MAT a	SNF11
YDR120C	MAT a	TRM1
YDR159W	MAT a	SAC3
YDR192 C	MAT a	NUP42
YDR395W	MAT a	SXM1
YDR410C	MAT a	STE14
YDR458C	MAT a	HEH2
YDR532 C	MAT a	KRE28
YEL017W	MAT a	GTT3
YGL016W	MAT a	KAP122
YGL035C	MAT a	MIG1
YGL086W	MAT a	MAD1
YGL115W	MAT a	SNF4
YGL241W	MAT a	KAP114
YGR202 C	MAT a	PCT1
YGR212W	MAT a	SLI1
YHL020C	MAT a	OPI1
YHR076W	MAT a	PTC7
YHR134W	MAT a	WSS1
YHR195W	MAT a	NVJ1
YIL016W	MAT a	SNL1
YIL149C	MAT a	MLP2
YJL080C	MAT a	SCP160
YJL079C	MAT a	PRY1
YJL073W	MAT a	JEM1
YJL030W	MAT a	MAD2
YKL057C	MAT a	NUP120
YKL068W	MAT a	NUP100
YKR044W	MAT a	UIP5
YLL023 C	MAT a	POM33
YLR018C	MAT a	POM34
YLR262 C	MAT a	YPT6
YLR240W	MAT a	VPS34
YLR265C	MAT a	NEJ1
YLR335W	MAT a	NUP2
YML034W	MAT a	SRC1
YMR153W	MAT a	NUP53
YMR255W	MAT a	GFD1
YMR284W	MAT a	YKU70
YNL199C	MAT a	GCR2
YNL159C	MAT a	ASI2
YNL008C	MAT a	ASI3
YNL012W	MAT a	SPO1
YOR112W	MAT a	CEX1
YOR311 C	MAT a	DGK1
YOL072W	MAT a	THP1
YPL200W	MAT a	CSM4
YPL192 C	MAT a	PRM3
YPL186C	MAT a	UIP4
YPL125W	MAT a	KAP120
YLR064W	MAT a	PER33
YBR273 C	MAT a	UBX7
YCR045C	MAT a	RRT12
YCR086W	MAT a	CSM1
YKR082W	MAT a	NUP133
YLR450W	MAT a	HMG2
YER110C	MAT a	KAP123
YER120W	MAT a	SCS2
YER123W	MAT a	YCK3
YMR065W	MAT a	KAR5
YNR075W	MAT a	COS10
YDR424C	MAT a	DYN2
YML103 C	MAT a	NUP188

To determine nuclear envelope permeability during meiosis we utilized a nucleus-localized TetR-GFP fusion protein we previously reported [33].

The following alleles have been reported previously: *P_DMC1_-GFP-MPS3-NC, P_GPD1_-GAL4.ER*, and *P_GAL_-NDT80* [34,35]. The gene mutations and tagged alleles were first generated in *MATa* and *MATα* haploid cells; homozygous diploids (zygotes) were obtained by mating the corresponding haploids.

### Yeast culture methods

Yeast cells were grown in YPD (1% yeast extract, 2% peptone, and 2% dextrose) at 30°C. To induce meiosis, YPD cultures were diluted with YPA (1% yeast extract, 2% peptone, and 2% potassium acetate) to reach OD (optical density, λ = 600 nm) of 0.2 and incubated at 30^°^C for approximately 14 h to reach a final OD of ~1.6–1.8. Yeast cells were then washed once in water and resuspended in 2% potassium acetate to induce meiosis. Cells were allowed to grow for approximately 6 h before microscopy to ensure that the majority of cells had initiated meiosis II. The onset of meiosis II is referred to as time zero in our time-lapse microscopy experiments.

To induce the expression of the GAL promoter in meiosis, 2 μM β-estradiol (final concentration) was added to a portion of the culture medium 6 h after transferring to 2% potassium acetate. An equal volume of ethanol was added to the control strains. To initiate the degradation of AID tagged proteins, 2 mM 1-napthylene acetic acid (final concentration) was added to a portion of the culture medium 6 h after transferring to 2% potassium acetate. An equal volume of ethanol was added to the control strains. Cultures were shaken vigorously at 30^°^C and protein extracts collected at designated time points prior to time-lapse microscopy.

### Time-lapse fluorescence microscopy

Prior to microscopy, yeast cells were prepared as described previously [33]. Briefly, an agarose pad with 2% potassium acetate was prepared on a concave slide, and a small aliquot of yeast cells were placed on the agarose, sealed with a coverslip, and scoped for the desired time duration. Time-lapse fluorescence microscopy was carried out on a DeltaVision imaging system (GE Healthcare Life Sciences) at 30°C. We used a 60x (NA = 1.40) objective lens on an inverted microscope (IX-71, Olympus). Microscopic images were acquired with a CoolSNAP HQ2 charge-coupled device camera (Photometrics). Pixel size was set at 0.10700 μm. Time intervals were set at 5 min, and optical sections at 12, each with 0.5 μm thickness. Ultra-high signal-to-background coated custom filter sets were used. For GFP, the excitation spectrum was at 470/40 nm, emission spectrum at 525/50 nm; for RFP, excitation was at 572/35 nm, and emission at 632/60 nm. To minimize photo toxicity to the cells and photo bleaching to fluorophores, we used neutral density filters to limit excitation light to 32% or less of the normal equipment output.

### Microscopy data analysis

Acquired microscopy images were deconvolved using the SoftWorx package (GE Healthcare Life Sciences). Projected images are used for figure display. To determine the fluorescence intensity of Pom34-GFP at the midzone and around the meiotic nuclei, we defined a 16 × 16 pixel area covering the entirety of the cell and obtained the total GFP fluorescence intensity from single optical sections. We then defined a 10 × 10 pixel area covering a single nucleus based on the Hta1-mApple signal and obtained the total GFP fluorescence intensity from single optical sections. The net percent intensity was determined by subtracting the background from each nucleus and the total GFP signal of the cell. The mean percentage of GFP localized to the nuclei of at least 25 cells was analyzed in each strain. Mutant phenotypes were analyzed similarly, or based on observed Hta1-mApple signal for cells with more than four masses. We used one-way ANOVA tests to determine statistical significance.

### Genetic screen to identify NPC interacting factors

From the yeast deletion collection (ATCC-GSA-5), a pooled *MATa* library containing deletions of individual open reading frames that encode known nuclear-envelope-associated proteins was used to screen the localization of Pom34-GFP. The library was crossed to a yeast strain containing Pom34-GFP and Hta1-mApple. After mating, meiosis was induced in diploids by resuspending in 2% potassium acetate. Resulting tetrads were dissected, genotyped, and corresponding homozygous zygotes were generated. Yeast cells were induced to undergo meiosis in 2% potassium acetate and visualized periodically for progression through meiosis II with a 100× objective lens (NA = 1.40) mounted on a motorized epifluorescence microscope (AxioImager, Zeiss).

### Protein extraction and western blotting

Yeast aliquots were withdrawn at indicated times for protein extraction with the trichloroacetic acid (TCA) method as described previously [36]. Briefly, 4 mL of yeast cells were collected, resuspended in 2.5% ice cold TCA, and then incubated at 4^°^C for 10 min. Cell pellets were stored at −80^°^C, and proteins were extracted by bead beating with a mini bead-beater homogenizer for 90 seconds at 4^°^C. Pom34-GFP was detected by an anti-GFP mouse monoclonal antibody (1:10 K dilution, Thermo Fisher Scientific, cat#GF28R). Vps4-V5-AID was detected by an anti-V5 antibody (1:5 K, Thermo Fisher Scientific, cat#R960-25). The level of Pgk1 was probed by a Pgk1 antibody (Thermo Fisher Scientific, cat#PA5-28,612) to serve as a loading control. Horseradish peroxidase-conjugated secondary antibodies, goat anti-mouse (Bio-Rad, cat#1,706,516), were used to probe the proteins of interest by an enhanced chemiluminescence (ECL) kit (Bio-Rad, cat#1,705,060). The ChemiDoc MP Imaging System (Bio-Rad, cat#17,001,402) was used to detect the ECL-based western blot. To calculate the relative protein abundance at each time point, individual band intensities were measured using the IPLab Imaging Software in conjunction with an in-house GelAnalyzer script and exported to Microsoft Excel. Target protein band intensities were made relative to those of the loading control (Pgk1).

### Replicative lifespan analysis

Lifespan analysis was carried out by micromanipulation as described previously [37] with the following modification. Cells were grown to saturation in YPD at 30^°^C. To induce meiosis, YPD cultures were diluted and washed once in water. Cultures were then resuspended in 2% potassium acetate and incubated for 16 h at 30^°^C to ensure the completion of meiosis. These cells were digested with 2% glusulase at 4^°^C for about 12 hr and plated on YPD plates where tetrads were identified and each gamete was isolated. After each cell division of the gamete, the daughter cell was removed and the number of generations was recorded. At least 15 tetrads, totaling 60 spores, were used for each experiment. Cells were grown at 30^°^C and stored overnight at 4^°^C. Every cell was followed until cell lysis was observed.

## Results

### Nucleoporins are sequestered during meiosis II in budding yeast

During our investigation of the meiotic nuclear envelope in budding yeast, we observed that Nup49, an FG-nucleoporin of the NPC central core [38], aggregated to the mid plane of a dividing anaphase II nucleus (Figure 1a and see below). By live-cell fluorescence microscopy, we found similarly that Pom34, a subunit of the transmembrane ring of the NPC [39], aggregated like Nup49 Figures 1b and 1c. Our findings therefore confirm that nucleoporins are sequestered during meiosis II, leading to the formation of the fifth nuclear component [30,31], which is also called GUNC for gametogenesis uninherited nuclear compartment [40].

To visualize Pom34 and Nup49, we generated alleles with their encoding proteins tagged C-terminally with either GFP or mApple Figures 1b and 1c. The expression of these alleles was under the control of their endogenous promoters, and they served as the only functional copies in the yeast genome. We used histone H2A, one of the two copies encoded by *HTA1*, fused to mApple to serve as a marker for the nucleus, allowing us to determine meiotic cell progression on the basis of nuclear morphology. By time-lapse live-cell fluorescence microscopy, we found that Pom34-GFP was sequestered to the GUNC during anaphase II and was largely excluded from the newly forming nuclei Figure 1b. We also found the fusion protein, TetR-GFP, which harbors a nuclear localization signal, was retained inside the nucleus throughout meiosis, with no discernable GFP signal present in the cytoplasm (Supplemental Figure 1). Therefore, sequestration of Pom34 to GUNC does not impair nuclear trafficking during meiosis.

To confirm that nucleoporin aggregation is not limited to certain NPC subcomplexes, we analyzed Pom34-GFP and Nup49-mApple together and observed their colocalization throughout meiosis, resulting in a change in positioning from along the nuclear periphery to the GUNC compartment Figure 1c. We also observed the aggregation of nucleoporins Nup170 and Pom152, as shown previously [31]. We therefore conclude that nucleoporins, including Nup49, Nup170, Pom34 and Pom152, are sequestered to GUNC and are largely excluded from the developing nuclei after anaphase II.

### Sequestration to the GUNC compartment is selective to nucleoporins

To determine if non-NPC proteins at the nuclear envelope are sequestered to GUNC, we used the INM protein Mps3 and ONM protein Mps2 as representatives. We have shown previously that Mps3, also localized to the spindle pole body (SPB), is cleaved and degraded during yeast meiosis [35]. To stabilize Mps3, we used a meiosis-specific non-degradable *MPS3* allele, *P_DMC1_-GFP-MPS3-NC* [35]. As expected, GFP-Mps3-NC was concentrated at the SPB but also localized along the nuclear periphery throughout meiosis Figure 2a. Notably, Mps3-NC did not appear to be confined to the GUNC compartment during anaphase II like the nucleoporins Figure 2a.

Next, we determined the localization of Mps2 in budding yeast meiosis Figures 2b and 2c. Like Mps3, the ONM protein Mps2 is also concentrated at the SPB in addition to its localization to the nuclear periphery Figure 2c. Mps2-GFP is mostly detectable as puncta corresponding to the SPB during meiosis (our unpublished data). In order to examine Mps2 distribution along the nuclear periphery, we generated a conditional *P_GAL_-GFP-MPS2* allele to effectively overexpress *MPS2* in meiosis Figure 2b. We used the *GAL4.ER P_GAL_-NDT80* constructs [34], henceforth called *P_GAL_-NDT80*, to arrest yeast cells at prophase I, and upon the addition of β-estradiol, release the yeast cells from prophase I arrest, therefore achieving highly synchronized yeast cultures undergoing meiosis II. In cells containing *P_GAL_-GFP-MPS2 P_GAL_-NDT80*, the addition of β-estradiol also led to the overproduction of Mps2 in meiosis II Figures 2b and 2c. We found that GFP-Mps2 localized to both the SPB and the nuclear periphery Figure 2c. Notably, Mps2 behaved similarly to Mps3, distributing along the periphery throughout the GUNC compartment and also along the nascent gamete nuclei, indicating that both inner and outer nuclear membranes are present at the GUNC.

In addition, we observed an even distribution of INM-localized Heh1 throughout the nuclear periphery and the GUNC (Supplemental Figure 2 and [31]. Taken together, our findings indicate that nucleoporins, but not any NE-associated proteins, are selectively sequestered to the GUNC compartment.

### Heh1 and Heh2 play a key role in nucleoporin sequestration

To identify factors that regulate nucleoporin sequestration, we used a genetic approach. From the yeast deletion collection library (ATCC-GSA-5), we pooled together 83 deletions of nonessential yeast genes that encode nuclear-envelope-associated proteins and determined their effect on Pom34-GFP confinement during anaphase II (Figure 3a and Supplemental Table 4). From this genetic screen, we found that yeast cells without *HEH1*, which is known to function in NPC assembly and surveillance in budding yeast [21], failed to retain Pom34-GFP to the GUNC Figure 3b. In the absence of Heh1, Pom34-GFP still aggregated during early meiosis II, but was not confined to GUNC; instead it became visible at the newly forming nuclei prior to the completion of meiosis Figures 1b and 3b). *HEH1* has a paralog, *HEH2*; we, therefore, examined the role of Heh2 in nucleoporin sequestration by analyzing Pom34-GFP in *heh2Δ* cells Figure 3c. On the basis of fluorescence intensity, we estimated the percentage of Pom34-GFP on the newly forming nuclei compared to the total detectable cellular Pom34-GFP Figure 3d. In *heh1Δ* cells the distribution of Pom34-GFP to the newly forming nuclei showed approximately 1.5-fold increase compared to the wild type Figure 3d. Importantly, the *heh1Δ heh2Δ* double mutant showed a 2-fold increase of Pom34-GFP distributed to the newly forming nuclei Figure 3d, demonstrating that Heh1 and Heh2 are partially redundant in constricting nucleoporins to GUNC. In addition, we found that removal of Heh2 caused another striking phenotype, with Pom34-GFP circling a single Hta1-mApple mass Figure 3c. The appearance of Pom34-GFP encapsulating a single Hta1-mApple mass occurred in about 68% of the *heh2Δ* cells Figure 3e. Together, our findings demonstrate that Heh1, and to a lesser degree Heh2, plays a role in confining nucleoporins to the GUNC during meiosis II.

### The ESCRT-III component Chm7 regulates nucleoporin sequestration

Heh1 is known to function together with the ESCRT-III component Chm7 in NPC assembly and surveillance in vegetative yeast cells [24,25]; we therefore hypothesized that Chm7 is required for nucleoporin sequestration in meiosis. Using time-lapse live-cell microscopy, we observed that in *chm7Δ* cells, the accumulation of Pom34-GFP in the GUNC took place during anaphase II, however sequestration from the developing nuclei was severely hindered, with over 50% of the Pom34-GFP associating with the nascent nuclei Figures 4a and 4e. These findings suggest that the ESCRT-III component Chm7 is necessary for maintaining GUNC.

To determine whether Chm7 and Heh1 act together in nucleoporin sequestration, we examined Pom34-GFP distribution in *heh1Δ chm7Δ* double mutant cells Figure 4b. We found that Pom34-GFP was not confined to the GUNC, with 73% of the total Pom34-GFP signal distributed along the developing prospore nuclei Figure 4e, demonstrating that the lack of Pom34-GFP sequestration in the *heh1Δ chm7Δ* double mutant is more severe than in either the *chm7Δ* or *heh1Δ* single mutant Figure 4e. Because Heh1 is thought to recruit Chm7 to the nuclear envelope [25], the observed synergistic effect of Chm7 and Heh1 on Pom34-GFP distribution is unexpected and raises the possibility that additional factors from ESCRT-III are required for sequestration of nucleoporins to GUNC (see below). Alternatively, there may also be redundancies within the GUNC formation pathway that are capable of confining nucleoporins.

Recent work has shown that Spo21, an outer plaque component of the SPB, is required for nucleoporin sequestration [31]. In the absence of Spo21, sequestration of nucleoporins to GUNC became defective Figure 4c and [31]. To determine the epistatic relationship between *SPO21* and *CHM7*, we examined Pom34-GFP localization in *spo21Δ chm7Δ* double mutant cells Figure 4d. Removal of Spo21 and Chm7 simultaneously did not significantly change the distribution of Pom34-GFP along the nascent nuclei, indicating that Spo21 acts upstream of Chm7 Figure 4e. We note that removal of Heh1 or Chm7, individually or simultaneously, did not alter the protein level of Pom34 Figures 4f, 4g and 4i, demonstrating that Heh1 and Chm7 regulate nucleoporin localization, but not protein stability. In contrast, removal of Spo21 altered Pom34 protein level, and mature spores never formed (Figure 4h and our unpublished data). This is likely due to the requirement of Spo21 in meiotic cell progression and spore formation [41,42]. Taken together, our findings suggest that *SPO21* acts epistatically to *CHM7* to sequester nucleoporins to the GUNC.

### The ESCRT-III-associated factor Vps4 regulates nucleoporin sequestration

Vps4, which associates with the ESCRT-III complex, has been shown to function with Chm7 during NPC surveillance in budding yeast [24]. We hypothesized that Vps4 is required for nucleoporin sequestration in meiosis. First, we determined Vps4 localization with a *VPS4-GFP* allele, which served as the only functional copy of *VPS4* in the budding yeast genome Figure 5a. In meiosis II, Vps4 formed multiple foci inside the cell Figure 5a. Approximately one discernible Vps4 focus was localized to the GUNC and attached to each nucleus Figure 5a, demonstrating that Vps4 is associated with GUNC and the meiotic nuclei. Next, we generated a conditional *vps4-AID* allele to deplete Vps4 specifically after the induction of meiosis Figures 5b-5d, because deletion of *VPS4* prevented budding yeast cells from undergoing meiosis (our unpublished data). With *P_GAL_-NDT80*, yeast cells were arrested at prophase I, and addition of 1-napthylacetic acid (NAA) led to the degradation of Vps4-AID Figures 5b-5d. Cells were then released from prophase I with the addition of β-estradiol to activate *P_GAL_-NDT80* and allowed to proceed through meiosis II in the absence of Vps4. We found that depletion of Vps4 in meiosis resulted in defects in nucleoporin sequestration and Hta1-mApple partitioning in over 90% of the cells observed Figures 5e-5i. The most common mutant phenotype observed in cells depleted of Vps4 was similar to that of the *chm7Δ* and *heh1Δ* mutants, in which Pom34-GFP was no longer confined to the GUNC Figure 5i. On the basis of the Pom34-GFP fluorescence intensity, we characterized meiotic cells in four categories: type 1, wild type, with less than 50% of the total Pom34-GFP presenting on the nascent nuclei; type 2, with over 50% of the total Pom34-GFP presenting on the nascent nuclei; type 3, with Pom34-GFP encapsulating one of the four Hta1-mApple masses; and type 4, with Pom34-GFP encapsulating a fifth Hta1-mApple mass Figures 5e-I. As expected, wild-type and *vps4-AID* cells without NAA treatment showed mostly the type 1 phenotype, 81% and 74%, respectively, Figures 5e and 5i. Upon depletion of Vps4-AID with NAA, we observed Pom34-GFP dispersed along the nuclear periphery, type 2, in 46% of cells Figures 5f and 5i. The accumulation of Pom34-GFP on a single Hta1-mApple mass, a phenotype similar to that of *heh1Δ* and *heh2Δ* mutants (type 3), was also observed in 34% of Vps4-depleted cells Figures 5g and 5i. Finally, we observed the formation of a 5th Hta1-mApple mass encapsulated by Pom34-GFP (type 4) occurred in about 18% of Vps4-depleted cells Figures 5h and 5i. We found that the *vps4-AID heh1Δ* double mutant showed an increase in the frequency of type 2 cells compared to the single mutants, with 58% of *vps4-AID heh1Δ* cells lacking Pom34-GFP sequestration Figure 5i. However, the percentage of type 3 and type 4 cells appeared comparable in the double mutant Figure 5i. Together, our findings demonstrate that the ESCRT-III factor Vps4 is required for nucleoporin sequestration during meiosis II.

### ESCRT-III and its associated factors regulate gamete replicative lifespan

To determine whether ESCRT-III-mediated nuclear envelope remodeling regulates gamete rejuvenation, we determined the replicative lifespan of gametes derived from mutants that impaired GUNC formation. Wild-type gametes had a maximal replicative lifespan of about 32 generations, with an average of 22 Figure 6. In contrast, the replicative lifespan of gametes derived from *heh1Δ* and *chm7Δ* mutants reduced to 15 generations on average Figure 6, demonstrating the importance of Heh1 and Chm7 in restoring replicative potential. Importantly, gametes from the *heh1Δ chm7Δ* mutant reduced the maximal replicative lifespan by nearly half, with an average replicative lifespan of 10 generations Figure 6. Similarly, depletion of Vps4 via the *vps4-AID* allele, reduced the replicative lifespan of its gametes Figure 6. Of note, the *vps4-AID* allele permitted us to deplete Vps4 specifically in meiosis Figures 5b-5d, thus eliminating a potential deleterious effect the removal of mitotic Vps4 may exert on replicative lifespan during the vegetative growth of the gamete. Taken together, these findings suggest that Heh1 and ESCRT-III are critical for maintaining gamete replicative lifespan.

## Discussion

In this study, we confirm that nucleoporins are sequestered during meiosis II in budding yeast. Importantly, we show the LEM-domain protein Heh1 and the ESCRT-III complex act in concert to confine nucleoporins and regulate gamete rejuvenation. Our findings not only are consistent with a recent work showing the sequestration of NPCs and other age-induced damages away from the newly-formed gametes [30,31], but also provide a mechanistic understanding for the process of GUNC and its potential role in rejuvenation of gametes in budding yeast, which may have implications for understanding how self-renewal is achieved during animal gametogenesis.

### How is GUNC formed and maintained in meiosis II?

We have shown that nucleoporins, but not any nuclear-envelope-associated proteins, are selectively sequestered to the GUNC. In their recent work, King et al. have shown that other age-induced factors, including extrachromosomal rDNA circles and heat shock proteins, also are aggregated in the GUNC [31], indicating that formation of the GUNC is a coordinated process involving the nuclear envelope and the nuclear plasma.

All nucleoporins we tested are sequestrated to the GUNC during anaphase II, suggesting that the NPC as a whole, not just individual nucleoporins, is sequestered. One exception is Nup2, the nucleoporin that binds to the cytoplasmic or nucleoplasmic surface of the NPC, that appears to not be confined at the GUNC. Instead, Nup2 is distributed evenly along the nuclear periphery and also potentially associated with the ER (our unpublished data and [31], which distribution is likely mediated by the ability of Nup2 to associate and dissociate with the NPC depending on the level of Ran-GTP present in the cell [43,44].

**Figure 7.**
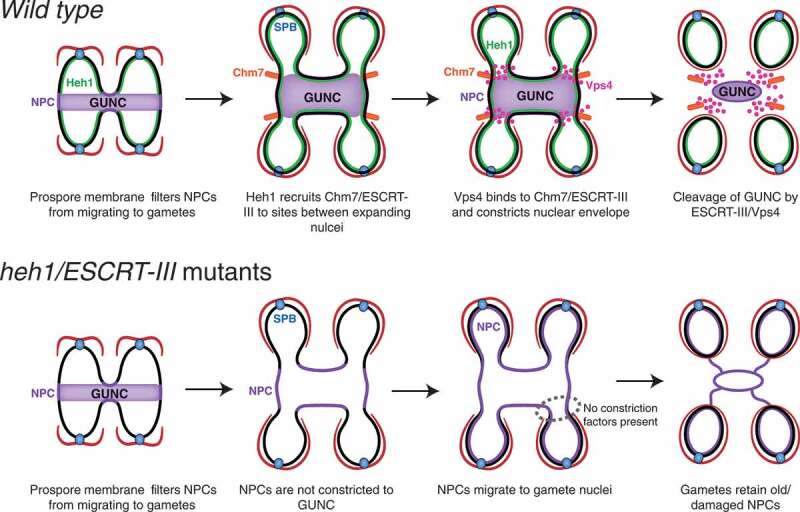
Model for ESCRT-III-mediated GUNC formation in budding yeast meiosis. Top, GUNC formation and maintenance in wild type. Note that NPCs are sequestered away from the newly formed gamete nuclei. Bottom, GUNC fails to form in the absence of Heh1/ESCRT-III function at the nuclear envelope. The GUNC compartment is shown in purple; Chm7/ESCRT-III in orange. Pink dots represent Vps4; red lines prospore membrane. NPC, nuclear pore complex; SPB, spindle pole body.

How then is GUNC formed? Previous work has shown that the SPB meiotic outer plaque component Spo21 is required for nucleoporin sequestration to GUNC [40]. In the absence of Spo21, nucleoporins largely spread out throughout the nuclear periphery without any evidence of aggregation. Spo21 and its associated outer plaque are uniquely modified on the cytoplasmic side of the SPB during meiosis to form a membrane-organizing center that is necessary for establishing the prospore membrane, which encircles the four gametes at the end of meiosis [28]. We speculate that newly formed prospore membrane, emanating from the SPB, actively ‘pushes’ NPCs to the GUNC Figure 7. Alternatively, rapid expansion of the nuclear membranes during anaphase II, which coincides with the formation of the prospore membrane, passively leaves the old NPCs behind in the GUNC. In this regard, the newly formed prospore membranes act as a selective filter, facilitating the constriction of old NPCs and hindering migration into the gamete nuclei. Our finding that ESCRT-III is required for sequestering nucleoporins to the GUNC lends support to this confinement model Figure 7. Furthermore, Spo21 appears to act epistatically to ESCRT-III (this study), indicating that ESCRT-III-mediated GUNC functions in the context of prospore membrane biogenesis.

### How does ESCRT-III constrict NPCs to GUNC?

We have shown that the ESCRT-III component Chm7 and the AAA-ATPase Vps4, a cofactor of ESCRT-III, are crucial for restricting nucleoporins to the GUNC. Functions of the highly conserved ESCRT machinery revolve around membrane fission reactions throughout the cell [45,46]. In addition, upon the completion of mitosis when the nuclear envelope reforms in animal cells, ESCRT-III/Vps4 facilitates sealing of nuclear membrane fenestrations [47–49]. We speculate that during late anaphase II, the LEM-domain protein Heh1 acts as a site-specific adaptor to recruit ESCRT-III, for example Chm7, to the sites between the expanding nuclei and the GUNC. Vps4 then likely binds to the ESCRT-III complex and constricts the nuclear envelope, resulting in the cleavage of the GUNC compartment from the newly formed nuclei Figure 7. This reasoning is consistent with the observation that *HEH1*, but not its paralog *HEH2*, is upregulated in late meiosis [50]. Furthermore, Vps4 forms foci at the junctions of the GUNC and the expanding prospore nuclear envelope (this study). In the absence of ESCRT-III function, NPCs would move freely from the GUNC without restriction to the expanding daughter nuclei Figure 7. This reasoning is analogous to nuclear division in budding yeast mitosis, in which the mother cell retains all of the detrimental morphological and physiological changes causing the mother cell to age [3,4]. By way of concerted actions from Heh1 and ESCRT-III, the GUNC thus functionally resembles the mother cell in mitosis and restricts the old or damaged NPCs and other nuclear senescence factors from segregating into the newly formed gametes Figure 7. Because Heh1 directs ESRCT-III during NPC biogenesis by exposure to the cytoplasm and subsequent binding of Chm7 [25], the activity of Heh1 may hold the key to where and how the GUNC constriction sites are determined. Because NPC sequestration is not completely abolished in the absence of ESCRT-III function, ESCRT-independent pathways could also be at work. Alternatively, NPCs may be passively left behind when the prospore nuclear membrane expands rapidly outward from the spindle pole body during late meiosis Figure 7. Without ESCRT-III, certain NPCs may fortuitously be trapped in the GUNC.

### ESCRT-III-mediated GUNC regulates gamete rejuvenation

We show that ESCRT-III and its associated factors help maintain the replicative lifespan of gametes. Removal of meiotic ESCRT-III activity diminishes the replicative lifespan of gametes in budding yeast (this study). We speculate that one of the major functions of the GUNC is to restrict old or damaged NPCs from being incorporated into the newly formed gametes. NPCs are known to function as both barrier and gateway between the cytoplasm and nucleoplasm, ensuring cellular homeostasis between compartments, and the disruption of this barrier has been shown in several different contexts, including a variety of human diseases [51]. Alternatively, ESCRT-III-mediated NPC confinement may not be directly modulating aging but instead serve as a readout of cellular aging similar to other unwanted materials such as protein aggregates and extrachromosomal rDNA circles. In either case, sequestration of NPCs to the GUNC in budding yeast meiosis represents a means for nuclear quality control and provides a new paradigm for elucidating the mechanism of cellular aging.

We note that the nuclear envelope appears to be intact and its permeability remains normal in meiosis II when the GUNC occurs. Although budding yeast undergoes closed meiosis and mitosis, the GUNC process in meiosis functionally mimics the nuclear envelope breakdown seen in higher eukaryotes, which permits the removal of nuclear senescence factors. Indeed, in fission yeast at anaphase II, the nuclear envelope undergoes a ‘virtual’ breakdown, during which the permeability of the nuclear envelope is temporally increased [52,53]. In that regard, the GUNC process may represent a primordial version of nuclear envelope remodeling that precedes nuclear envelope breakdown. Future studies on the regulation of ESCRT-III-mediated GUNC formation and maintenance in budding yeast will provide further insight into the functionality of the GUNC and cellular rejuvenation.

## Supplementary Material

Supplemental Material
