# Supplementary material for: The ESCRT-III complex is required for nuclear pore complex sequestration and regulates gamete replicative lifespan in budding yeast meiosis
Source: Nucleus. 2020 Sep 6;11(1):219–36. doi: 10.1080/19491034.2020.1812872 (PMC7529410; doi:10.1080/19491034.2020.1812872)
Supplement: Supplemental Material [file KNCL_A_1812872_SM4596.zip › Supplementary information/Supplemental legends.docx]

**Supplemental Information**

Supplemental Figure 1. Nuclear envelope integrity is maintained throughout meiosis II**.** Time-lapse live-cell microscopy showing the localization of TetR-NLS-GFP, which harbors a nuclear localization signal, during meiosis II. Time 0 refers to the perceived start point of meiosis II based on nuclear morphology as shown in Fig 1B. Hta1-mApple marks the yeast histone H2A. Projected images from 12 optical sections are shown.

Supplemental Figure 2. The INM protein Heh1 is not confined to the GUNC. Time-lapse live-cell microscopy showing the localization of Heh1-GFP during meiosis II. Time 0 refers to the perceived start point of meiosis II based on nuclear morphology. Projected images from 12 optical sections are shown.

Supplemental Table 1. Yeast stains used in this study.

Supplemental Table 2. PCR primers used in this study.

Supplemental Table 3. Plasmids used in this study.

Supplemental Table 4. List of gene deletions used in genetic screen
