# Supplementary material for: The ESCRT-III complex is required for nuclear pore complex sequestration and regulates gamete replicative lifespan in budding yeast meiosis
Source: Nucleus. 2020 Sep 6;11(1):219–36. doi: 10.1080/19491034.2020.1812872 (PMC7529410; doi:10.1080/19491034.2020.1812872)
Supplement: Supplemental Material [file KNCL_A_1812872_SM4596.zip › Supplementary information/Supplemental-tables.docx]

Table 1. Yeast strains used in this study

| **Strain** | **Background** | **Genotype** | **Experiment** |
| --- | --- | --- | --- |
| HY6229 | SK1 | *MATa/MATα, his3∆200, leu2-k, ura3, lys2, ho::LYS2, POM34-GFP//his3∆200, leu2-k, ura3, lys2, ho::LYS2, POM34-GFP, HTA1-mApple* | Figures 1B, 4E, 4F, 4G, 4I, and 6 |
| HY6279 | SK1 | *MATa/MATα, his3∆200, leu2-k, ura3, lys2, ho::LYS2, POM34-GFP, NUP49-mApple//his3∆200, leu2-k, ura3, lys2, ho::LYS2, POM34-GFP, NUP49-mApple* | Figure 1C |
| HY6200 | SK1 | *MATa/MATα, his3∆200, leu2-k, ura3, lys2, ho::LYS2, HTA1-mApple//his3∆200, leu2-k, ura3, lys2, ho::LYS2, P_DMC1_-MPS3-NC* | Figure 2A |
| HY6695 | SK1 | *MATa/MATα, ho::LYS2, lys2, ura3, leu::hisG, his3::hisG, trp1::hisG, P_GAL_-NDT80::TRP1, ura3::P_GPD1_-GAL4.ER::URA3, P_GAL1_-GFP-Mps2// ho::LYS2, lys2, ura3, leu::hisG, his3::hisG, trp1::hisG, P_GAL_-NDT80::TRP1, ura3::P_GPD1_-GAL4.ER::URA3, P_GAL1_-GFP-Mps2, HTA1-mApple* | Figures 2B and 2C |
| HY6586-2 | SK1 | *MATa/MATα, his3∆200, leu2-k, ura3, lys2, ho::LYS2, heh1∆::KAN, POM34-GFP//his3∆200, leu2-k, ura3, lys2, ho::LYS2, heh1∆::KAN, POM34-GFP, HTA1-mApple* | Figures 3B, 3D, 3E, 4E, and 6 |
| HY6356 | SK1 | *MATa/MATα, his3∆200, leu2-k, ura3, lys2, ho::LYS2, heh2∆::KAN, POM34-GFP//his3∆200, leu2-k, ura3, lys2, ho::LYS2, heh2∆::KAN, POM34-GFP, HTA1-mApple* | Figures 3C, 3D, and 3E |
| HY6389 | SK1 | *MATa/MATα, his3∆200, leu2-k, ura3, lys2, ho::LYS2, heh1∆::KAN, heh2∆::KAN, POM34-GFP, HTA1-mApple//his3∆200, leu2-k, ura3, lys2, ho::LYS2, heh1∆::KAN, heh2∆::KAN, POM34-GFP, HTA1-mApple* | Figures 3D and 3E |
| HY6541 | SK1 | *MATa/MATα, his3∆200, leu2-k, ura3, lys2, ho::LYS2, chm7∆::HB, POM34-GFP//his3∆200, leu2-k, ura3, lys2, ho::LYS2, chm7∆::HB, POM34-GFP, HTA1-mApple* | Figures 4A, 4E, 4F, 4I, and 6 |
| HY6585 | SK1 | *MATa/MATα, his3∆200, leu2-k, ura3, lys2, ho::LYS2, heh1∆::KAN, chm7∆::HB, POM34-GFP, HTA1-mApple//his3∆200, leu2-k, ura3, lys2, ho::LYS2, heh1∆::KAN, chm7∆::HB, POM34-GFP, HTA1-mApple* | Figures 4B, 4E, 4G, 4I, and 6 |
| HY6608 | SK1 | *MATa/MATα, his3∆200, leu2-k, ura3, lys2, ho::LYS2, spo21∆::KAN, POM34-GFP//his3∆200, leu2-k, ura3, lys2, ho::LYS2, spo21∆::KAN, POM34-GFP, HTA1-mApple* | Figures 4C, 4E, 4H, and 4I |
| HY6614 | SK1 | *MATa/MATα, his3∆200, leu2-k, ura3, lys2, ho::LYS2, spo21∆::KAN, chm7∆::HB, POM34-GFP//his3∆200, leu2-k, ura3, lys2, ho::LYS2, spo21∆::KAN, chm7∆::HB, POM34-GFP, HTA1-mApple* | Figures 4D, 4E, 4H, and 4I |
| HY6724 | SK1 | *MATa/MATα, his3Δ200, leu2-k, ura3, lys2, ho::LYS2, P_DMC1_-HEH1-mApple, VPS4-GFP::HIS5//his3Δ200, leu2-k, ura3, lys2, ho::LYS2, P_DMC1_-HEH1-mApple, VPS4-GFP::HIS5* | Figure 5A |
| HY6663 | SK1 | *MATa/MATα, ho::LYS2, lys2, ura3, leu::hisG, his3::hisG, trp1::hisG, P_GAL_-NDT80::TRP1, ura3::P_GPD1_-GAL4.ER::URA3, P_DMC1_-TIR1::LEU2, VPS4-V5-AID::HIS5, POM34-GFP, HTA1-mApple// ho::LYS2, lys2, ura3, leu::hisG, his3::hisG, trp1::hisG, P_GAL_-NDT80::TRP1, ura3::P_GPD1_-GAL4.ER::URA3, P_DMC1_-TIR1::LEU2, VPS4-V5-AID::HIS5, POM34-GFP, HTA1-mApple* | Figures 5B, 5C, 5D, 5F, 5G, 5H, 5I and 6 |
| HY6644 | SK1 | *MATa/MATα, ho::LYS2, lys2, ura3, leu::hisG, his3::hisG, trp1::hisG, P_GAL_-NDT80::TRP1, ura3::P_GPD1_-GAL4.ER::URA3, POM34-GFP, HTA1-mApple// ho::LYS2, lys2, ura3, leu::hisG, his3::hisG, trp1::hisG, P_GAL_-NDT80::TRP1, ura3::P_GPD1_-GAL4.ER::URA3, POM34-GFP, HTA1-mApple* | Figures 5B, 5E and 5I |
| HY6705 | SK1 | *MATa/MATα, ho::LYS2, lys2, ura3, leu::hisG, his3::hisG, trp1::hisG, P_GAL_-NDT80::TRP1, ura3::P_GPD1_-GAL4.ER::URA3, P_DMC1_-TIR1::LEU, VPS4-V5-AID, heh1∆::KAN, POM34-GFP, HTA1-mApple// ho::LYS2, lys2, ura3, leu::hisG, his3::hisG, trp1::hisG, P_GAL_-NDT80::TRP1, ura3::P_GPD1_-GAL4.ER::URA3, P_DMC1_-TIR1::LEU2, VPS4-V5-AID, heh1∆::KAN, POM34-GFP, HTA1-mApple* | Figures 5B, 5F, 5G, 5H and 5I |

Table 2. Primers used in this study

| **Primer Name** | **Sequence** |
| --- | --- |
| POM34-tagF | GCAAATATGCATATATGATGAACTCACAGTCCCCAAGGGGGAAAATAGCGGCCGCTCTAGAACTAGT |
| POM34-tagR | TATATAGCTATGGAAAGTATTAAATGTTTTTTTGCTGTTTTCCCCCTCGAGGTCGACGGTA |
| HTA1-tagF | GTTGCCAAAGAAGTCTGCCAAGGCTACCAAGGCTTCTCAAGAATTAGCGGCCGCTCTAGAACTAGTGG |
| HTA1-tagR | GCAGTTTAGTTCCTTCCGCCTTCTTTAAAATACCAGAACCGATCTCCCCCTCGAGGTCGACGGTATCG |
| NUP49-tagF | GTTACATCAAAAAACGAAAACACTGGCATCATTGAGCATAGCGGCCGCTCTAGAACTAGT |
| NUP49-tagR | ACTTGTTATACGCACTATATAAACTTTCAGGGCGATTTACCCCCCTCGAGGTCGACGGTA |
| HEH1-deletionF | TCACCCTGAACGGAAATCAA |
| HEH1-deletionR | TTTCTTTCCTCCATGTGTCG |
| HEH2-deletionF | TGACAAGCACTATCTTCCAAAGT |
| HEH2-deletionR | GTATGCGTAGGGGAAGGGAT |
| HEH1-tagF | TCGATGAAAAGGTTAAACCGCAGATCCCGCAGTTACGGAAAtCAGGGGCATGATGTGACT |
| HEH1-tagR | TTCCGCCAATGTTGTTGTCAGTGGGTGATAACTAGAGAAAGCTCGTTTTCGACACTGGAT |
| CHM7-deletionF | AGTGCAGCGTTAGTAGAGACAATAAGAGGAGTTTTAAATTCTTAAACAGGGGCATGATGTGACT |
| CHM7-deletionR | TGCACAGGTCCTTCATTTGTATTTATCTTCAGATTATTCAATCTCTTTAATAGCTCGTTTTCGACACTGGAT |
| SPO21-deletionF | TCTGGGTTCAAGAATTCCTCAGA |
| SPO21-deletionR | GTTTCTTCGGCAACCCTGTA |
| VPS4-tagF | CTTGCTGAAGCAAGAACAGTTCACTAGAGAGATTTTGGTCAAGAAGGTAACGCGGCCGCTCTAGAACTAGTGG |
| VPS4-tagR | TATTTTCATGTACACAAGAAATCTACATTAGCACGTTAATCAATTGACCCCTCGAGGTCGACGGTATCG |

Table 3. Plasmids used in this study

| **Plasmid Name** | **Description** |
| --- | --- |
| pHG363 | *P_DMC1_-GFP-MPS3-NC, LEU2* |
| pHG527 | *P_GAL1_-GFP-MPS2, LEU2* |
| pHG496 | *P_GAL1_-HEH1, LEU2* |
| pHG273 | *P_DMC1_-TIR1, URA3* |
| pHG740 | *P_DMC1_-TIR1, LEU2* |
| pHG742 | *P_DMC1_-HEH1-mApple, LEU2* |

Table 4. Gene deletions used in genetic screen

| Systematic name | Ploidy | Standard name |
| --- | --- | --- |
| YML107C | MAT a | PML39 |
| YMR129W | MAT a | POM152 |
| YHR004C | MAT a | NEM1 |
| YER027C | MAT a | GAL83 |
| YDR205W | MAT a | MSC2 |
| YBR150C | MAT a | TBS1 |
| YIL030C | MAT a | SSM4 |
| YAL009W | MAT a | SPO7 |
| YAR002W | MAT a | NUP60 |
| YAR027W | MAT a | UIP3 |
| YAR042W | MAT a | SWH1 |
| YAR044W | MAT a | SWH1 |
| YBL079W | MAT a | NUP170 |
| YBR097W | MAT a | VPS15 |
| YBR170C | MAT a | NPL4 |
| YDL019C | MAT a | OSH2 |
| YDL088C | MAT a | ASM4 |
| YDL089W | MAT a | NUR1 |
| YDL116W | MAT a | NUP84 |
| YDR073W | MAT a | SNF11 |
| YDR120C | MAT a | TRM1 |
| YDR159W | MAT a | SAC3 |
| YDR192C | MAT a | NUP42 |
| YDR395W | MAT a | SXM1 |
| YDR410C | MAT a | STE14 |
| YDR458C | MAT a | HEH2 |
| YDR532C | MAT a | KRE28 |
| YEL017W | MAT a | GTT3 |
| YGL016W | MAT a | KAP122 |
| YGL035C | MAT a | MIG1 |
| YGL086W | MAT a | MAD1 |
| YGL115W | MAT a | SNF4 |
| YGL241W | MAT a | KAP114 |
| YGR202C | MAT a | PCT1 |
| YGR212W | MAT a | SLI1 |
| YHL020C | MAT a | OPI1 |
| YHR076W | MAT a | PTC7 |
| YHR134W | MAT a | WSS1 |
| YHR195W | MAT a | NVJ1 |
| YIL016W | MAT a | SNL1 |
| YIL149C | MAT a | MLP2 |
| YJL080C | MAT a | SCP160 |
| YJL079C | MAT a | PRY1 |
| YJL073W | MAT a | JEM1 |
| YJL030W | MAT a | MAD2 |
| YKL057C | MAT a | NUP120 |
| YKL068W | MAT a | NUP100 |
| YKR044W | MAT a | UIP5 |
| YLL023C | MAT a | POM33 |
| YLR018C | MAT a | POM34 |
| YLR262C | MAT a | YPT6 |
| YLR240W | MAT a | VPS34 |
| YLR265C | MAT a | NEJ1 |
| YLR335W | MAT a | NUP2 |
| YML034W | MAT a | SRC1 |
| YMR153W | MAT a | NUP53 |
| YMR255W | MAT a | GFD1 |
| YMR284W | MAT a | YKU70 |
| YNL199C | MAT a | GCR2 |
| YNL159C | MAT a | ASI2 |
| YNL008C | MAT a | ASI3 |
| YNL012W | MAT a | SPO1 |
| YOR112W | MAT a | CEX1 |
| YOR311C | MAT a | DGK1 |
| YOL072W | MAT a | THP1 |
| YPL200W | MAT a | CSM4 |
| YPL192C | MAT a | PRM3 |
| YPL186C | MAT a | UIP4 |
| YPL125W | MAT a | KAP120 |
| YLR064W | MAT a | PER33 |
| YBR273C | MAT a | UBX7 |
| YCR045C | MAT a | RRT12 |
| YCR086W | MAT a | CSM1 |
| YKR082W | MAT a | NUP133 |
| YLR450W | MAT a | HMG2 |
| YER110C | MAT a | KAP123 |
| YER120W | MAT a | SCS2 |
| YER123W | MAT a | YCK3 |
| YMR065W | MAT a | KAR5 |
| YNR075W | MAT a | COS10 |
| YDR424C | MAT a | DYN2 |
| YML103C | MAT a | NUP188 |
